# Combining host immune response biomarkers and clinical scores for early prediction of sepsis in infection patients

**DOI:** 10.1080/07853890.2024.2396569

**Published:** 2024-08-30

**Authors:** Xiaoming Zhou, Chen Liu, Zhe Xu, Jiaze Song, Haijuan Jin, Hao Wu, Qianhui Cheng, Wenqian Deng, Dongyuan He, Jingwen Yang, Jiaying Lin, Liang Wang, Zhiyi Wang, Chan Chen, Jie Weng

**Affiliations:** a Department of General Practice, The Second Affiliated Hospital and Yuying Children’s Hospital of Wenzhou Medical University, Wenzhou, China; b Wenzhou Key Laboratory of Precision General Practice and Health Management, Wenzhou, China; c Department of Geriatric Medicine, The First Affiliated Hospital, Wenzhou Medical University, Wenzhou, China; d Department of Intensive Care Unit, The Second Affiliated Hospital and Yuying Children’s Hospital of Wenzhou Medical University, Wenzhou, China; e The Second Clinical Medical College, Wenzhou Medical University, Wenzhou, China; f Theorem Clinical College of Wenzhou Medical University, Wenzhou Central Hospital, Wenzhou, China; g Taishun County People’s Hospital Medical Community Sixi Branch, Taishun, China; h Department of General Practice, Taizhou Women and Children’s Hospital of Wenzhou Medical University, Taizhou, China; i Department of Public Health, Robbins College of health and Human Sciences, Baylor University, Waco, TX, USA; j South Zhejiang Institute of Radiation Medicine and Nuclear Technology, Wenzhou, China

**Keywords:** Sepsis, infection, sepsis development, intensive care unit, mortality, immune response biomarkers, IL-10, IL-6, NEWS, SIRS

## Abstract

**Background:**

The performance of host immune responses biomarkers and clinical scores was compared to identify infection patient populations at risk of progression to sepsis, ICU admission and mortality.

**Methods:**

Immune response biomarkers were measured and NEWS, SIRS, and MEWS. Logistic and Cox regression models were employed to evaluate the strength of association.

**Results:**

IL-10 and NEWS had the strongest association with sepsis development, whereas IL-6 and CRP had the strongest association with ICU admission and in-hospital mortality. IL-6 [HR (95%CI) = 2.68 (1.61–4.46)] was associated with 28-day mortality. Patient subgroups with high IL-10 (≥ 5.03 pg/ml) and high NEWS (> 5 points) values had significantly higher rates of sepsis development (88.3% vs 61.1%; *p* < 0.001), in-hospital mortality (35.0% vs. 16.7%; *p* < 0.001), 28-day mortality (25.0% vs. 5.6%; *p* < 0.001), and ICU admission (66.7% vs. 38.9%; *p* < 0.001).

**Conclusions:**

Patients exhibiting low severity signs of infection but high IL-10 levels showed an elevated probability of developing sepsis. Combining IL-10 with the NEWS score provides a reliable tool for predicting the progression from infection to sepsis at an early stage. Utilizing IL-6 in the emergency room can help identify patients with low NEWS or SIRS scores.

## Introduction

Sepsis, which is a dysregulated immune response to infection resulting in organ dysfunction [1,2], remains a major challenge in healthcare. Despite notable advancements in sepsis treatment in recent years, the occurrence of sepsis in infected patients greatly increases the risk of mortality and organ dysfunction [3–5]. Therefore, it is vital to accurately assess the severity of the host’s response and the likelihood of disease progression in order to effectively manage sepsis and decrease mortality rates.

Early antibiotic administration is linked to improved survival in sepsis, emphasizing the clinical importance of early screening and detection [6]. There is currently a lack of effective biomarkers or tools to assist clinicians in assessing patients with potentially high progression of disease following low severity scores from assessment tools such as the National Early Warning Score (NEWS) [7], Modified Early Warning Score (MEWS) [8], or Systemic Inflammatory Response Syndrome (SIRS) scores [9]. This lack of clarity can have a significant impact on treatment decisions. Early Goal-Directed Therapy (EGDT) is commonly initiated following a sepsis diagnosis, and implemented aggressive treatment strategies in patients with low risk of sepsis progression could increase treatment costs and potentially lead to complications [10]. Conversely, delaying treatment in high-risk sepsis developed patients may result in higher mortality rates and more ICU admissions. Therefore, accurately determining the severity of infection-related conditions and early pathophysiological changes caused by host responses is crucial for making optimal decision-making and reducing ICU admissions.

Although several risk factors predicting sepsis patient outcomes have been identified, very few studies have determined the progression of infection to sepsis. Consequently, there are currently no clinically validated tools to guide clinicians in making treatment decisions. Given the pathophysiological hallmark of sepsis characterized by the dysregulation of host immune responses, some studies have reported alterations in host immune response biomarkers early in the infection, which hold value in prognosticating sepsis. However, whether these markers can accurately identify the potential for early infection patients to progress to sepsis remains unclear. Recent studies [11,12] have suggested that host immune response markers may enhance the performance of NEWS, MEWS, or SIRS scores to identify the progression or mortality.

Therefore, this study seeks to evaluate whether the integration of host immune response biomarkers and scoring systems can reliably estimate the risk of in­fection patients progressing to sepsis and mortality, aiming to provide clinicians with a comprehensive, accurate tool to assess patient risk and guide treatment strategies.

## Methods

### Study population

**Figure 1.**
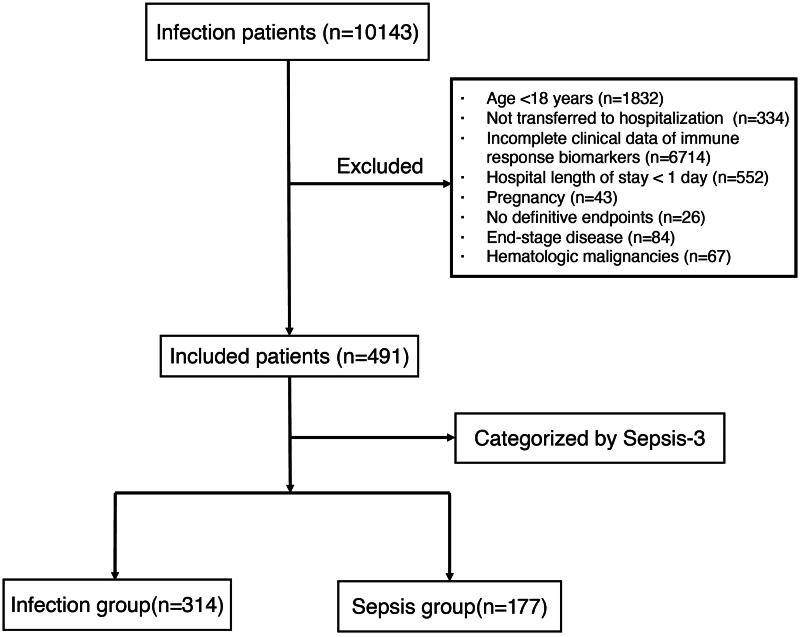
Flowchart of patient selection.

This retrospective observational cohort study included adult patients admitted to the emergency and hospitalized for infectious diseases at a local large tertiary hospital located in Southern China from January 2019 to December 2021. The details of recruitment process are illustrated in Figure 1. Inclusion criteria were as follows: (1) Definitive infection; (2) No-sepsis upon emergency department admission; (3) Age over 18 years. Exclusion criteria were: (1) Pregnancy; (2) Not transferred to hospitalization; (3) Missing data for immune response biomarkers; (4) Hospital length of stay < 1 day; (5) Unable to determine endpoints; (6) Terminal stage of disease or at end of life; (7) Hematologic malignancies. Infection was judged based on usual clinical practice, and according to vital signs, main symptoms, or laboratory findings during emergency department, as determined by two clinicians with over 5 years of experience each. The diagnosis of sepsis was specifically determined by these same clinicians. The definition of sepsis was based on the criteria set forth by the Third International Consensus Definitions for Sepsis and Septic Shock (Sepsis-3) as the presence of infection or suspected infection, accompanied by an acute increase in the total Sequential Organ Failure Assessment (SOFA) score of at least 2 points [1]. The study was approved by the institutional review board of the Second Affiliated Hospital of Wenzhou Medical University (NO. 2021-k-18-01, approval date: March 5, 2021, study title: Combining Host Immune Response Biomarkers and Clinical Scores for Early Prediction of Sepsis in Infection Patients). This study was conducted in accordance with the ethical standards of the responsible committees on human experimentation and with the Helsinki Declaration of 1975. This project was approved by the Ethics Committee of the Second Affiliated Hospital of Wenzhou Medical University. Due to the retrospective nature of the study, the requirement for informed consent was waived. Further details about enrollment, demographical and clinical variables are provided in the Additional File

### Data collection

Demographic characteristics, comorbidities, initial vital signs at the time of emergency department admission, source of infection, laboratory tests and prognoses, were collected through the electronic medical record system (EMRS). Laboratory parameters indicators in­cluded: (1) Immune response biomarkers: lymphocyte subsets: CD3, CD4, CD8, CD4/CD8, CD16 + 56, and CD19; Th1/Th2 subset cytokines: interleukin-2 (IL-2), interleukin-4 (IL-4), interleukin-6 (IL-6) and interleukin-10 (IL-10), tumor necrosis factor-alpha (TNF-α) and interferon-gamma (IFN-γ), as well as complement series (C3 and C4) and immunoglobulin series (IgA, IgE, IgM and IgG); (2) Routine laboratory indicators: C-reactive protein (CRP), procalcitonin (PCT), leukocyte count, liver and kidney function, coagulation function, etc.; (3) Clinical scores: NEWS, SIRS, and MEWS. For patients with a hospital stay of less than 28 days, survival status on the 28th day following emergency department presentation was determined through phone.

### Outcome

The primary endpoint was the progression of infection to sepsis after hospitalization. The secondary endpoints were in-hospital mortality, established by the survival situation of the patient upon their discharge from the hospital, 28-day mortality, and ICU admission.

### Statistical analysis

Normally distributed variables were reported using mean and standard deviation values, and skewed data were reported using median, and interquartile range. Outliers were detected using residual examination. Demographic and clinical data were assessed using the chi-square(χ^2^) or Fisher’s exact test for categorical variables, and either symmetrical or skewed continuous variables were assessed using analysis of variance (ANOVA) or the Mann-Whitney U test.

The predictive ability of biomarkers and clinical scores with primary outcomes was assessed using the receiver operating characteristics (ROC) and areas under the curve (AUC), with 95% confidence intervals (95% CI) used to assess significance. Optimal cut-off values for sensitivity and specificity were assessed using Youden’s criterion, and subgroups determined according to optimal cut-off values for the prediction of 28-day mortality. Kaplan-Meier curves were used to visualize the temporal distribution of mortality among patients stratified by the optimal 28-day cutoff and further classify subgroups stratified by the optimal cutoff. All patient groups were compared by the log-rank test.

Univariate and multivariate logistic regression analysis assessed the association of each immune response biomarker, inflammatory response biomarker and clinical score with progression of infection, ICU admission and in-hospital mortality, and corresponding Cox regression analysis assessed the association with 28-day mortality. Multivariate analysis included the baseline clinical factors with significant differences (*p* < 0.05) between the two groups in the univariate analysis as adjusting variables. Results were presented as either the odds ratio (OR) or the hazard ratio (HR) per 1 interquartile-range increase for logistic and Cox regression analyses, respectively.

With the addition of the IL-10 to the inflammatory response biomarkers and clinical scores, to evaluate whether the accuracy of the prediction of infection progress would improve, the AUCs, continuous net reclassification improvement (NRI), and integrated discrimination improvement (IDI) were calculated.

A restricted cubic spline was used with three knots placed at the 25th, 50th, and 75th centiles to flexibly model the association between IL-6 and 28-day mortality. The 95th centile was used to minimize the influence of potential outliers. Non-linearity was assessed using a likelihood ratio test.

The statistics software R (version 3.4.3) were used for all statistical analysis. A *p* value < 0.05 was considered statistically significant.

## Results

### Patient characteristics

10143 patients were assessed for eligibility and 9652 excluded (mostly due to missing biomarker data). The final 491 adult infection patients were enrolled in our study (Figure 1). The main sources of infection were the pulmonary and abdominal regions. For pneumonia patients, radiological findings were positive, and patients with intra-abdominal infections also exhibited corresponding radiological changes. Among the entire cohort, 177 patients (36.0%) developed sepsis, and 39 patients (7.94%) developed septic shock. Patients who developed sepsis showed higher NEWS, SIRS, and MEWS scores compared with infected patients. More detailed baseline information was shown in Table 1.

**Table 1.** Patient baseline characteristics stratified by progression of infection to sepsis.

Variables	All (*N* = 491)	Infection (*N* = 314)	Sepsis (*N* = 177)	*p* Value
Demographics				
Age, years	65 [52–75]	62 [48–72]	69 [56–79]	<0.001
Male sex, n (%)	297 (60.5%)	185 (58.9%)	112 (63.3%)	0.394
Infection source				<0.001
Respiratory, n (%)	321 (65.4%)	200 (63.7%)	121 (68.4%)	
Intra-abdominal, n (%)	108 (22%)	83 (26.4%)	25 (14.1%)	
Urogenital, n (%)	36 (7.33%)	23 (7.32%)	13 (7.34%)	
Skin and soft tissue, n (%)	10 (2.04%)	5 (1.59%)	5 (2.82%)	
Unknow, n (%)	8 (1.63%)	2 (0.64%)	6 (3.39%)	
Others, n (%)	8 (1.63%)	1 (0.32%)	7 (3.95%)	
Antibiotic initiation time, min	95 [50–226]	120 [56–281]	68 [39–134]	<0.001
Clinical scores				
NEWS, points	2 [0–3]	1 [0–2]	3 [1–5]	<0.001
SIRS, points	1 [0–2]	1 [0–1]	2 [1–2]	<0.001
MEWS, points	1 [1–3]	1 [1–2]	2 [1–3]	0.016
Outcome				
In-hospital mortality, n (%)	42 (8.6%)	0 (0.00%)	42 (23.7%)	<0.001
28-day mortality, n (%)	29 (5.9%)	0 (0.00%)	29 (16.4%)	<0.001
Hospital LOS, days	8 [5–13]	7 [5–10]	12 [8–22]	<0.001
ICU admission, n (%)	97 (19.8%)	2 (0.64%)	95 (53.7%)	<0.001
ICU LOS, day	6.5 [3–16.8]	14.5 [13.8–15.2]	6.0 [3–17.2]	0.300
Shock, n (%)	39 (7.94%)	0 (0.00%)	39 (22%)	<0.001
Vital signs				
Temperature, °C	37.0 [36.6–37.5]	36.9 [36.5–37.3]	37.1 [36.7–37.8]	<0.001
Respiratory rate, bpm	20 [18–20]	20 [18–20]	20 [19–22]	<0.001
Heart rate, bpm	88 [78–101]	86 [76–99]	93 [81–106]	<0.001
SBP, mmHg	127 ± 21	128 ± 20	125 ± 23	0.108
DBP, mmHg	75 ± 13	76 ± 12	72 ± 13	<0.001
SpO_2,_ %	98 [97–100]	99 [98–100]	98 [95–99]	<0.001
Comorbidities				
Cardiovascular disease, n (%)	52 (10.6%)	22 (7.01%)	30 (16.9%)	0.001
Liver disease, n (%)	22 (4.48%)	4 (1.27%)	18 (10.2%)	<0.001
Kidney disease, n (%)	29 (5.91%)	3 (0.96%)	26 (14.7%)	<0.001
HIV, n (%)	2 (0.41%)	0 (0.00%)	2 (1.13%)	0.129
Hypertension, n (%)	155 (31.6%)	81 (25.8%)	74 (41.8%)	<0.001
Diabetes, n (%)	93 (18.9%)	48 (15.3%)	45 (25.4%)	0.008
Malignancy, n (%)	38 (7.74%)	20 (6.37%)	18 (10.2%)	0.181
Autoimmune disease, n (%)	9 (1.83%)	5 (1.59%)	4 (2.26%)	0.728
Lymphocyte subsets				
CD3, %	70.8 [62.5–79.6]	72.5 [65.6–80.0]	68.2 [55.1–76.6]	<0.001
CD4, %	41.4 [30.7–49.2]	43.2 [34.5–49.8]	36.7 [27.9–46.0]	<0.001
CD8, %	24.2 [18.1–31.4]	25.2 [19.4–31.4]	23.0 [15.4–31.4]	0.021
CD19, %	12.5 [7.9–17.6]	11.9 [7.8–16.1]	14.4 [8.4–21.1]	0.001
CD4/8, %	1.67 [1.04–2.56]	1.67 [1.03–2.54]	1.66 [1.05–2.56]	0.692
CD16/56, %	13.8 [7.9–22.9]	12.4 [7.9–21.2]	15.6 [8.1–26.7]	0.056
Th1/Th2 cytokines				
IL-2, pg/ml	3.46 [2.55–3.88]	3.50 [2.59–3.88]	3.39 [2.45–4.02]	0.926
IL-4, pg/ml	3.1 [2.05–3.86]	3.12 [2.11–3.83]	3.08 [2–3.97]	0.976
IL-6, pg/ml	17.5 [6.9–54.2]	13.7 [5.5–31.7]	37.6 [12.6–170]	<0.001
IL-10, pg/ml	5.01 [3.79–7.16]	4.44 [3.55–5.83]	6.83 [4.88–12.1]	<0.001
INF-γ, pg/ml	3.15 [2.24–3.9]	3.04 [2.03–3.7]	3.31 [2.55 4.17]	0.002
TNF-α, pg/ml	2.92 [2.32–3.38]	2.89 [2.32–3.3]	2.96 [2.33–3.46]	0.231
Complements				
C3, mg/ml	113 [92.4–135]	117 [99.2–136]	104 [79.4–129]	<0.001
C4, mg/ml	23.4 [18.2–29.8]	23.5 [17.6–29.4]	23.3 [18.7–31.3]	0.25
Immunoglobulins				
IgA, mg/dl	236 [161–318]	239 [156–314]	221 [168–323]	0.864
IgE, mg/dl	189 [47–395]	163 [40–382]	206 [83–445]	0.008
IgG, mg/dl	1300 [1060–1580]	1285 [1050–1568]	1310 [1070–1600]	0.557
IgM, mg/dl	78 [57–110]	80 [60–111]	76 [53–107]	0.141
Other biomarkers				
CRP, mg/L	40 [7.03–115]	20.3 [3.76–85.7]	80.4 [22.9–169]	<0.001
PCT, ng/L	0.21 [0.04–1.69]	0.09 [0.03–0.92]	0.6 [0.13–3.15]	<0.001
White blood cells, *10^9^/L	7.61 [5.39–11]	7.31 [5.37–10.2]	8.34 [5.56–13.1]	0.022
Platelet, *10^9^/L	210 [154–273]	228 [184–286]	153 [89–221]	<0.001
Hemoglobin, g/L	119 ± 23.7	124 ± 19.6	110 ± 27.4	<0.001
ALT, U/L	21 [13–36]	19 [13–33]	27 [15–44]	<0.001
AST, U/L	23 [17–38]	21 [16–29]	31 [19–60]	<0.001
Globulin, g/L	25.5 [22.5–29]	25.9 [22.7–29]	24.7 [21.5–29.1]	0.113
Bilirubin, μmol/L	11.6 [7.6–17.9]	10.7 [7.3–15.4]	14.3 [8.2–21.5]	<0.001
Albumin, g/L	36.8 ± 5.5	38.5 ± 4.8	33.7 ± 5.4	<0.001
Creatinine, μmol/L	66 [52–83]	63 [52–74]	76 [54–110]	<0.001
Fibrinogen, g/L	5.99 [4.92–8.21]	5.74 [4.83–7.52]	6.69 [5.23–9.39]	<0.001
PT, s	13.8 [13.1–14.8]	13.5 [12.9–14.4]	14.6 [13.6–16]	<0.001
APTT, s	40 [36.3–45.5]	39 [35.9–43.5]	42.8 [37.2–49.3]	<0.001
D-Dimer, mg/L	1.3 [0.5–3.13]	0.84 [0.37–1.76]	2.9 [1.3–5.68]	<0.001
BNP, pg/L	226 [72–1105]	119 [55–286]	1230 [290–3650]	<0.001
CTnI, μg/mL	0.01 [0–0.02]	0.01 [0–0.01]	0.02 [0–0.07]	<0.001

SBP, systolic blood pressure; DBP, diastolic blood pressure; IL, interleukin; INF, interferon; TNF, tumor necrotic factor; CRP, C-reactive protein; PCT, procalcitonin; ALT, alanine aminotransferase; AST, aspartate aminotransferase; PT, prothrombin time; APTT, active partial thromboplastin time; BNP, brain natriuretic peptide; cTnI, cardiac troponin I; NEWS, National Early Warning Score; SIRS, systemic inflammatory response syndrome; MEWS, Modified Early Warning Score; LOS, length of stay; ICU, intensive care unit.

### Outcomes within the total population

**Figure 2.**
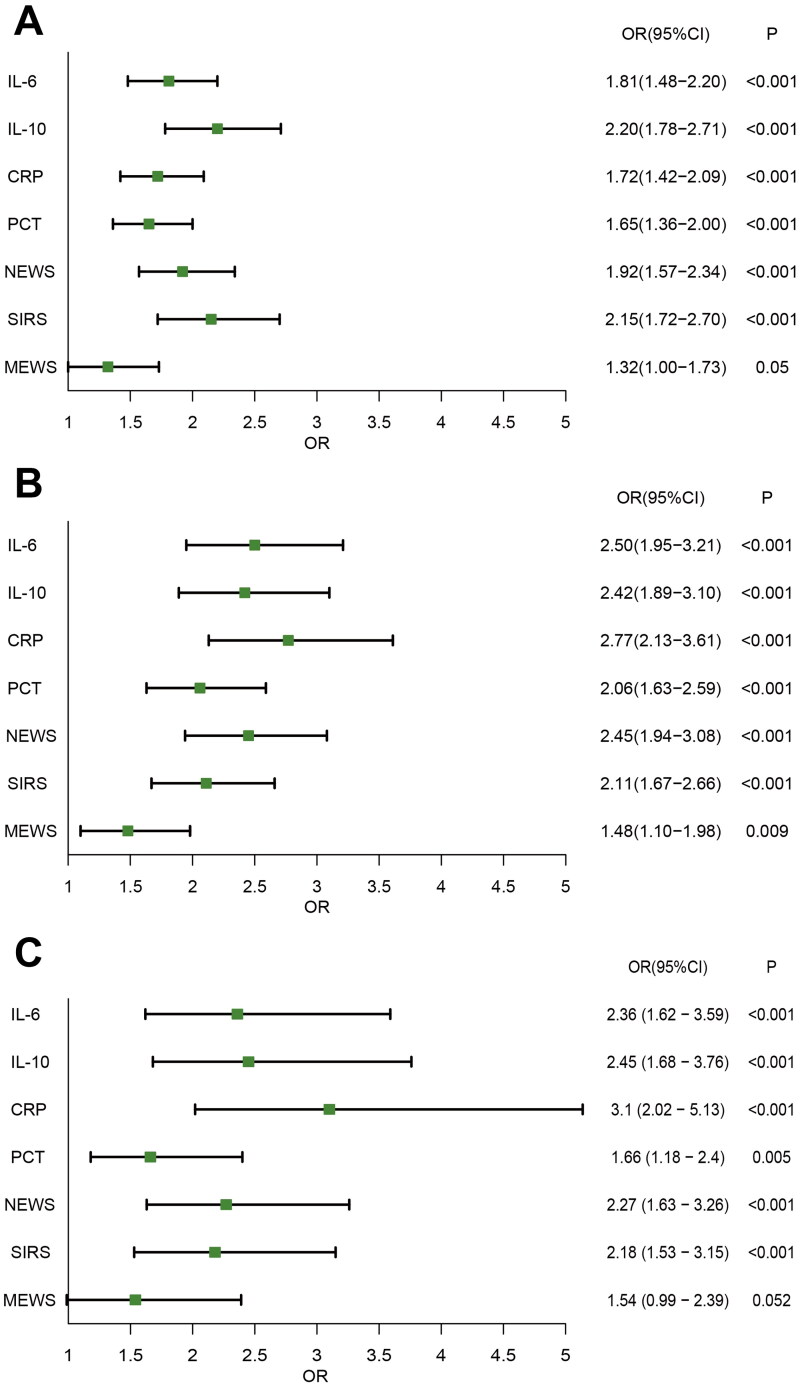
Multivariate logistic regression for progression of infection to sepsis (A), ICU admission (B) and in-hospital mortality (C).

The progression of infection to sepsis usually occurs within the first day, with the median time was 0 (0–1) day. Interestingly, patients who progressed to sepsis were started on antibiotics earlier (Table 1). Most biomarkers showed significant changes at the onset of infection in septic patients. Univariate logistic regression showed that IL-10 and NEWS had the strongest association with sepsis development (IL-10 vs. NEWS OR [95% CI] 2.22 [1.83–2.69] vs. 2.00 [1.66–2.41]; Table S1). The results of multivariate logistic regression analysis adjusted by age, hypertension, diabetes, cardiovascular disease, liver disease and renal disease variables showed that the association between IL-10 (OR [95% CI] 2.20 [1.78–2.71]) and NEWS (OR [95% CI] 1.92 [1.57–2.34]) remained consistent across sepsis development (Table S1; Figure 2A). Similar results could be found in corresponding AUROC analysis (Table S10; Figure S2A), with IL-10 having the optimal precision and NEWS having the greatest diagnostic odds ratio for progression of infection to sepsis. Compared with single indicator model, the addition of IL-10 into each of the other clinical scores and biomarkers significantly improved reclassification based on the NRI, IDI and significantly increased the AUC as the combined use of IL-10 and NEWS demonstrated the highest predictive capability (AUC = 0.789; Table S2).

A total of 97 patients (19.8%) required ICU admission. In addition, the 28-day mortality and in-hospital mortality rate were 5.9% (*N* = 29) and 8.6% (*N* = 42). IL-6 and IL-10, which have better predictive ability for sepsis among all immune response biomarkers, as well as CRP, PCT and clinical scores were selected for further analysis of their relationship with secondary endpoints. Univariate and multivariate Logistic regression found that IL-6 and CRP had the strongest association with ICU admission and in-hospital mortality (Tables S3 and S5; Figure 2B,C). Similar results could be found in corresponding AUROC analysis (Table S11 and S12; Figure S2B,D). The AUC significantly increased at both endpoints when IL-6 was combined with NEWS (Table S4 and S6). Univariate Cox regression analysis found that IL-6 [HR (95%CI) = 2.68 (1.61–4.46)] was associated with 28-day mortality (Table S7). In accordance with the results of multivariate Cox regression analysis adjusted, IL-6 kept strong association with 28-day mortality (Table S7). The AUROC analysis yielded concordant findings (Table S13; Figure S2C), wherein a threshold value of 65.58 pg/mL conferred the optimal prognostic odds ratio for 28-day mortality with the highest AUC (95% CI) of 0.839 (0.774–0.903). The Kaplan–Meier plot showed that IL-6 could accurately recognize low and high mortality patients compared to other biomarkers or scores (log-rank test *p* < 0.001; Figure S1A–F; Table S8).

The restricted cubic spline (Figure S3) delineated the association between serum IL-6 level and 28-day mortality, adjusted for covariates through multivariate Cox regression. IL-6 levels > 17.5 pg/mL exhibited a positive linear association with heightened risk of 28-day mortality (P_non-linear_ = 0.324).

### Subgroup analysis for sepsis development

The proportion of patients who developed sepsis was significantly (*p* < 0.01) higher in the high IL-10 and high NEWS (*N* = 53; 88.3%) as opposed to the low IL-10 and high NEWS (*N* = 11; 61.1%) subgroup (Table 2), with similar results also found for combinations of high IL-10 and SIRS (Table S14). Similar findings could also be found for IL-6 and NEWS subgroups (Table S15) or SIRS subgroups (Table S16).

**Table 2.** Patient subgroups stratified by IL-10 and NEWS.

Patient subgroups	IL-10	NEWS	IL-10	NEWS	IL-10	NEWS	IL-10	NEWS
	<5.03	<5	<5.03	≥5	≥5.03	<5	≥5.03	≥5
Population *N* (%)	229 (46.6%)		18 (3.7%)		184 (37.5%)		60 (12.2%)	
Sepsis *N* (%)	37 (16.2%)		11 (61.1%)		76 (41.3%)		53 (88.3%)	
Shock *N* (%)	5 (2.2%)		2 (11.1%)		19 (10.3%)		13 (21.7%)	
ICU admission *N* (%)	12 (5.2%)		7 (38.9%)		38 (20.7%)		40 (66.7%)	
28-day mortality *N* (%)	3 (1.3%)		1 (5.6%)		10 (5.4%)		15 (25.0%)	
Hospital mortality *N* (%)	3 (1.3%)		3 (16.7%)		15 (8.2%)		21 (35.0%)	
Hospital LOS, day	7 [5–10]		11 [5–17]		9 [6–13]		18 [7–29]	

IL, Interleukin; NEWS, National Early Warning Score; LOS, Length of Stay; ICU, intensive care unit.

### Subgroup analysis for ICU admission

Patients with l high IL-10 and high NEWS had a significantly higher risk of overall as well as ICU admission compared to corresponding patients with low IL-10 concentrations (*p* < 0.001) (Table 2). Similar results also found for combinations of high IL-10 and SIRS (Table S14). Similar subgroup enrichment could also be found in high IL-6 and NEWS subgroups (Table S15) or SIRS subgroups (Table S16).

### Subgroup analysis for 28-day mortality

**Figure 3.**
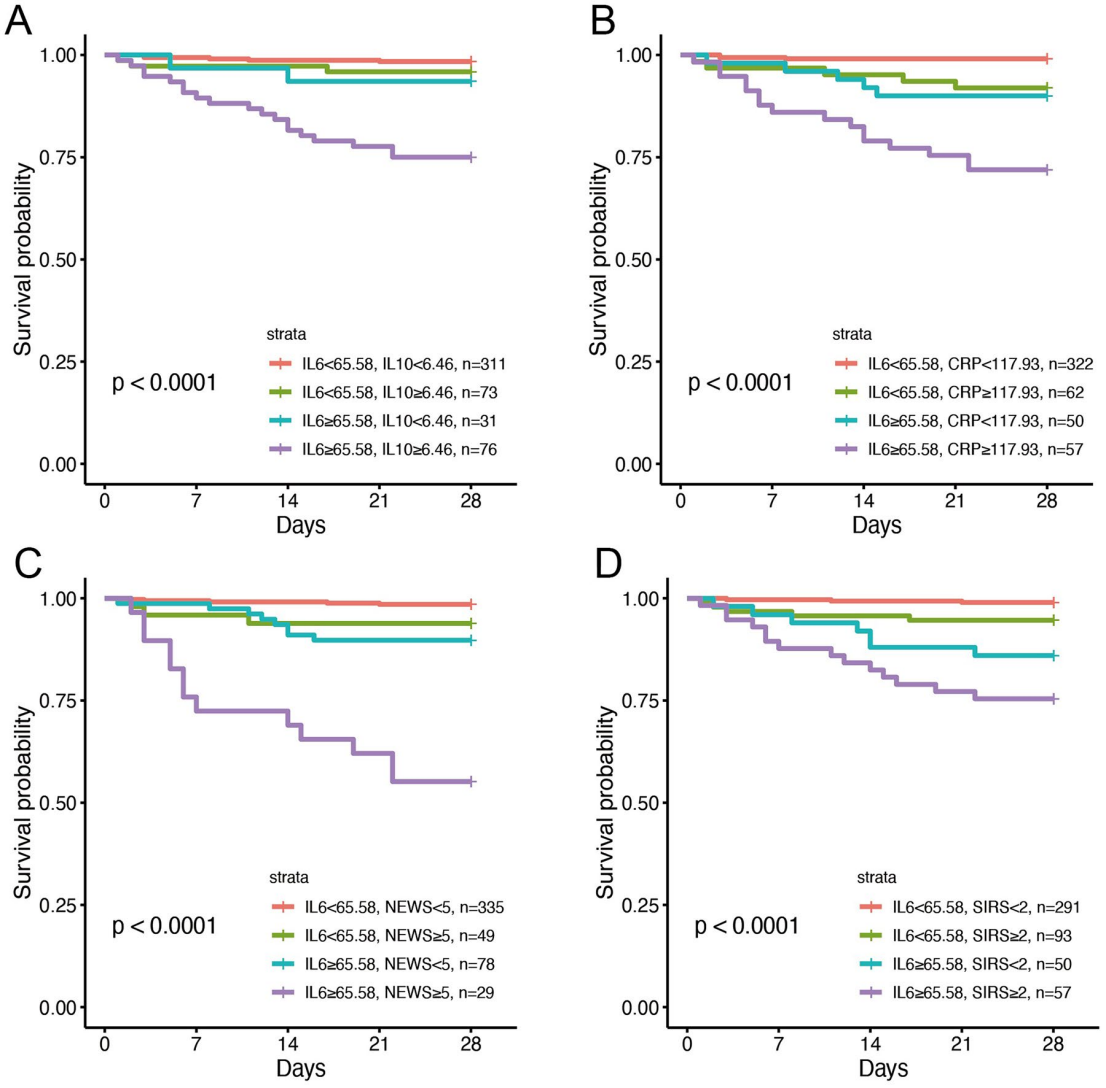
Kaplan–Meier curves for 28-day mortality according to a combination of IL-6 and IL-10 (A), CRP (B), NEWS (C) and SIRS (D).

For patients with low SIRS scores (< 2, *N* = 341), IL-6 was demonstrated to have the highest performance among all clinical scores or biomarkers in predicting 28-day mortality (*N* = 13; 3.1%; AUC = 0.859), followed by CRP and IL-10 (AUC = 0.771 and 0.762), indicating a comparable performance in relation to other clinical scores and biomarkers (Table S9). The presence of high IL-6 (≥ 65.58 pg/mL) and low SIRS (< 2) values resulted in a subgroup (*N* = 50; 10.2%) with instances of 28-day mortality (Figure 3D), while no such specialized subgroups (for example, low IL-10 [Figure 3A], low CRP [Figure 3B], and low NEWS [Figure 3C]) were found for any other clinical scores and biomarkers. There was no interaction between IL-6 and other biomarkers and clinical scores (Figure S4). In addition, patients stratified to the high IL-6 and high NEWS score (≥ 5) cohort exhibited the least favorable prognosis compared with the other patient cohorts (Figure 3C).

## Discussion

The current study demonstrates that immune response biomarkers possess predictive value in determining the occurrence of sepsis. Among these biomarkers, IL-10 exhibits the highest predictive capacity for the progression of infection to sepsis. Furthermore, the combination of IL-10 and NEWS score enhances the overall predictive capacity. Subgroup analysis reveals that patients with elevated IL-6 levels during infection face a greater risk of 28-day all-cause mortality, irrespective of the levels of other commonly used inflammation indicators or clinical scores.

Studies often focus on improving the accuracy of the sepsis prognosis, but studies focusing on the initiation, development, and early screening of sepsis are rare [13]. Serum CRP and PCT are currently the most frequently utilized indicators in clinical settings. However, their ability to predict sepsis early is somewhat limited [14–16]. Our study similarly discovered that the predictive capabilities of CRP and PCT for early sepsis were constrained. Sepsis is an outcome that arises from the interaction between pathogenic agents and host factors. The widely used quick SOFA (qSOFA) and SOFA scores have been shown to be effective in predicting sepsis mortality rates [17,18]. However, it is important to note that these scores are primarily used for evaluating disease severity, rather than for detecting and screening sepsis. As a result, monitoring dysregulated host-response biomarkers may offer valuable insights for the early identification of sepsis.

Various degrees of immune imbalance can be observed in sepsis patients including lymphocyte subgroups, Th1/Th2 subset cytokines, complement series, and immunoglobulin series et al. [19,20]. The 18 immune response biomarkers were analyzed in our study. These factors have been shown to have predictive value in determining the prognosis of sepsis patients. Zhu et al. found that the count of CD8+ T cells was predictive of the progression of sepsis and an association was observed between lymphopenia, CD8+ T cell depletion and the clinical outcomes of sepsis [21]. However, compared to Th1/Th2 subset cytokines, lymphocyte subsets are poor predictors of progression of infection to sepsis in this study.

Th1 cells primarily secrete IL-2, TNF-α, and IFN-γ, which predominantly mediate cellular immune responses. Th2 cells predominantly secrete IL-4, IL-6, and IL-10, which primarily mediate humoral immunity. Th1/Th2 subset cytokines express differently in response to infection may be relevant to sepsis [22,23]. Serum IL-6, as a pro-inflammatory cytokine, is correlated with immune cell recruitment, apoptosis, and complement activation in pathological processes. It shows an increase within 1–3 h after triggering factors, and it plays a role in recruiting acute-phase proteins and leukocytes for inflammatory clearance processes [24]. IL-10 is an anti-inflammatory cytokine that plays a crucial role in the development of sepsis. It acts by inhibiting the activity of macrophages and Th1 cells during infections, effectively suppressing the production of pro-inflammatory cytokines, polymorphonuclear cells, chemokines, eosinophils, and leukocytes, which prevents excessive damage to the host [25,26]. The excessive secretion of IL-6 and IL-10 indicates an early imbalance between pro- and anti-inflammatory res­ponses in patients with infection. Patients with a more severe imbalance are at a higher risk of progressing to sepsis, indicating a poorer prognosis. Davoudian et al. discovered that patients with sepsis had elevated concentrations of serum IL-1β, IL-6, IL-8, IL-10, IL-18, and TNF-α. These levels were even higher in patients with septic shock and were found to be correlated with a higher 90-day mortality rate [22]. Similar findings were observed in our study, indicating that patients with significantly elevated serum IL-6 and IL-10 concentrations at an early stage have the ability to predict progression to sepsis and ICU admissions.

Sepsis is highly heterogeneous, making valid predictions based on a single biomarker or clinical outcome difficult [27]. A meta-analysis evaluated 21 biomarkers and 7 clinical scores and showed that combining biomarkers with clinical scoring systems has better application value than using each biomarker alone [28] and Myrto Bolanaki et al. also found that PCT enhanced the early prediction of sepsis and offered additional value beyond the use of qSOFA alone [29]. However, few studies have combined host immune response markers and scoring systems to predict infection progression to sepsis. Zonneveld et al. [30] compared NEWS, SIRS, and qSOFA scores in predicting sepsis-related outcomes and found that the predictive value of NEWS score for deterioration of sepsis was better than that of SIRS and qSOFA scores. Similarly, the study by Almutary et al. [31] also showed that the NEWS score is a sensitive screening tool for predicting sepsis-related outcomes, but it lacks specificity. In this study, we discovered that IL-10 was the most effective predictor for progression from infection to sepsis and for assessing the prognosis of patients with infection. These findings were consistent with previous studies that demonstrated the usefulness of IL-10 in the early diagnosis of sepsis [32]. Additionally, the NEWS score exhibited the highest specificity. Furthermore, when IL-10 was combined with the NEWS score, the predictive ability for identifying the risk of infection patients progressing to sepsis was significantly improved, as evidenced by comparisons of the AUC, NRI, and IDI. The NRI, which is divided into three tiers: >0.6 (strong), 0.2–0.6 (medium), and <0.2 (weak) [33], showed that the combination of IL-10 with the NEWS score resulted in a medium incremental effect (0.555) compared to using IL-10 alone. Subgroups that combine IL-10 or IL-6 with NEWS or SIRS scores could further help identify high-risk individuals who may develop sepsis or require intensive care, allowing clinicians to further help guide treatment.

We then analyzed the relationship between immune response biomarkers and prognosis in infected patients and found that IL-6, CRP and NEWS index were significantly higher in survivors, consistent with previous studies [34]. IL-6 shows the strongest correlation with 28-day mortality, followed by CRP and IL-10. Kaplan-Meier curves showed that the high IL-6 group was significantly associated with higher 28-day all-cause mortality. Subgroup analysis showed that patients with high serum IL-6 levels may have worse clinical outcomes even if they have lower SIRS scores in the emergency department. This helps reduce the likelihood that emergency physicians will miss high-risk patients. The results of studies on patients with sepsis are consistent with our findings: the higher the level of IL-6 at the onset of sepsis, the worse the prognosis [35].

## Limitations

The present study has several limitations that should be acknowledged. Firstly, it is important to note that this study is a single-center retrospective observational study, which may introduce a certain degree of selection bias. Secondly, the sample size, especially within the sepsis group, is limited, and we excluded a significant number of patients due to missing immune response biomarkers, which could potentially lead to bias in the results. Additionally, our evaluation only focused on the baseline levels of indicators and clinical scores, without considering the impact of dynamic fluctuations in these indicators throughout the hospitalization period. Prospective studies are still needed in the future to validate our conclusions.

## Conclusion

The dysregulation of immune response biomarkers occurs in the early stages of infection. Combining IL-10 with the NEWS score provides a reliable tool for predicting the progression from infection to sepsis at an early stage. Utilizing IL-6 in the emergency room can help identify patients with low NEWS or SIRS scores who may be more susceptible to accepting less intensive treatment, but have a higher probability of a worse prognosis.

## Supplementary Material

Supplemental Material

## Data Availability

The datasets used and/or analysed during the present study are available from the corresponding author upon reasonable request.

## References

[1] Singer M, Deutschman CS, Seymour CW, et al. The third international consensus definitions for sepsis and septic shock (sepsis-3). JAMA. 2016;315(8):801–810. doi: 10.1001/jama.2016.0287. 26903338 PMC4968574

[2] Arı HF, Keskin A, Arı M, et al. Importance of lactate/albumin ratio in pediatric nosocomial infection and mortality at different times. Future Microbiol. 2024;19(1):51–59. doi: 10.2217/fmb-2023-0125. 37962487

[3] Rhee C, Chiotos K, Cosgrove SE, et al. Infectious diseases society of America position paper: recommended revisions to the national severe sepsis and septic shock early management bundle (SEP-1) sepsis quality measure. Clin Infect Dis. 2021;72(4):541–552. doi: 10.1093/cid/ciaa059. 32374861 PMC8189682

[4] Uffen JW, Oosterheert JJ, Schweitzer VA, et al. Interventions for rapid recognition and treatment of sepsis in the emergency department: a narrative review. Clin Microbiol Infect. 2021;27(2):192–203. doi: 10.1016/j.cmi.2020.02.022. 32120030

[5] Keskin A, Aci R. Procalcitonin to albumin ratio as a biomarker for predicting mortality in sepsis. J Coll Physicians Surg Pak. 2024;34(3):360–363. doi: 10.29271/jcpsp.2024.03.360. 38462876

[6] Mellhammar L, Linder A, Tverring J, et al. NEWS2 is superior to qSOFA in detecting sepsis with organ dysfunction in the emergency department. J Clin Med. 2019;8(8):1128. doi: 10.3390/jcm8081128. 31362432 PMC6723972

[7] Corfield AR, Lees F, Zealley I, et al. Utility of a single early warning score in patients with sepsis in the emergency department. Emerg Med J. 2014;31(6):482–487. doi: 10.1136/emermed-2012-202186. 23475607

[8] Subbe CP, Kruger M, Rutherford P, et al. Validation of a modified early warning score in medical admissions. QJM. 2001;94(10):521–526. doi: 10.1093/qjmed/94.10.521. 11588210

[9] Kaukonen KM, Bailey M, Pilcher D, et al. Systemic inflammatory response syndrome criteria in defining severe sepsis. N Engl J Med. 2015;372(17):1629–1638. doi: 10.1056/NEJMoa1415236. 25776936

[10] Rowan KM, Angus DC, Bailey M, et al. Early, goal-directed therapy for septic shock – a patient-level meta-analysis. N Engl J Med. 2017;376(23):2223–2234. doi: 10.1056/NEJMoa1701380. 28320242

[11] Backes Y, van der Sluijs KF, Mackie DP, et al. Usefulness of suPAR as a biological marker in patients with systemic inflammation or infection: a systematic review. Intensive Care Med. 2012;38(9):1418–1428. doi: 10.1007/s00134-012-2613-1. 22706919 PMC3423568

[12] Gonzalez Del Castillo J, Wilson DC, Clemente-Callejo C, et al. Biomarkers and clinical scores to identify patient populations at risk of delayed antibiotic administration or intensive care admission. Crit Care. 2019;23(1):335. doi: 10.1186/s13054-019-2613-4. 31665092 PMC6819475

[13] Weng J, Wu H, Xu Z, et al. The role of propionic acid at diagnosis predicts mortality in patients with septic shock. J Crit Care. 2018;43:95–101. doi: 10.1016/j.jcrc.2017.08.009. 28863283

[14] Leticia Fernandez-Carballo B, Escadafal C, MacLean E, et al. Distinguishing bacterial versus non-bacterial causes of febrile illness – a systematic review of host biomarkers. J Infect. 2021;82(4):1–10. doi: 10.1016/j.jinf.2021.01.028. 33610683

[15] Opal SM, Wittebole X. Biomarkers of infection and sepsis. Crit Care Clin. 2020;36(1):11–22. doi: 10.1016/j.ccc.2019.08.002. 31733673

[16] Bilgin M, Aci R, Keskin A, et al. Evaluation of the relationship between procalcitonin level and the causative pathogen in intensive care patients with sepsis. Future Microbiol. 2023;18(13):875–883. doi: 10.2217/fmb-2023-0010. 37594461

[17] Wu C, Ma J, Yang H, et al. Interleukin-37 as a biomarker of mortality risk in patients with sepsis. J Infect. 2021;82(3):346–354. doi: 10.1016/j.jinf.2021.01.019. 33545167

[18] Ling H, Chen M, Dai J, et al. Evaluation of qSOFA combined with inflammatory mediators for diagnosing sepsis and predicting mortality among emergency department. Clin Chim Acta. 2023;544:117352. doi: 10.1016/j.cca.2023.117352. 37076099

[19] Martin MD, Badovinac VP, Griffith TS. CD4 T cell responses and the sepsis-induced immunoparalysis state. Front Immunol. 2020;11:1364. doi: 10.3389/fimmu.2020.01364. 32733454 PMC7358556

[20] Arens C, Bajwa SA, Koch C, et al. Sepsis-induced long-term immune paralysis–results of a descriptive, explorative study. Crit Care. 2016;20(1):93. doi: 10.1186/s13054-016-1233-5. 27056672 PMC4823837

[21] Tang Y, Wu J, Tian Y, et al. Predictive value of peripheral lymphocyte subsets for the disease progression in patients with sepsis. Int Immunopharmacol. 2023;117:109922. doi: 10.1016/j.intimp.2023.109922. 37012888

[22] Davoudian S, Piovani D, Desai A, et al. A cytokine/PTX3 prognostic index as a predictor of mortality in sepsis. Front Immunol. 2022;13:979232. doi: 10.3389/fimmu.2022.979232. 36189302 PMC9521428

[23] Hotchkiss RS, Karl IE. The pathophysiology and treatment of sepsis. N Engl J Med. 2003;348(2):138–150. doi: 10.1056/NEJMra021333. 12519925

[24] Zhang Y, Li B, Ning B. Evaluating IL-6 and IL-10 as rapid diagnostic tools for Gram-negative bacteria and as disease severity predictors in pediatric sepsis patients in the intensive care unit. Front Immunol. 2022;13:1043968. doi: 10.3389/fimmu.2022.1043968. 36544765 PMC9760793

[25] Couper KN, Blount DG, Riley EM. IL-10: the master regulator of immunity to infection. J Immunol. 2008;180(9):5771–5777. doi: 10.4049/jimmunol.180.9.5771. 18424693

[26] Zhang W, Wang W, Hou W, et al. The diagnostic utility of IL-10, IL-17, and PCT in patients with sepsis infection. Front Public Health. 2022;10:923457. doi: 10.3389/fpubh.2022.923457. 35937269 PMC9355284

[27] Weng J, Hou R, Zhou X, et al. Development and validation of a score to predict mortality in ICU patients with sepsis: a multicenter retrospective study. J Transl Med. 2021;19(1):322. doi: 10.1186/s12967-021-03005-y. 34325720 PMC8319895

[28] Tong-Minh K, Welten I, Endeman H, et al. Predicting mortality in adult patients with sepsis in the emergency department by using combinations of biomarkers and clinical scoring systems: a systematic review. BMC Emerg Med. 2021;21(1):70. doi: 10.1186/s12873-021-00461-z. 34120605 PMC8201689

[29] Bolanaki M, Winning J, Slagman A, et al. Biomarkers improve diagnostics of sepsis in adult patients with suspected organ dysfunction based on the quick sepsis-related organ failure assessment (qSOFA) score in the emergency department. Crit Care Med. 2024;52(6):887–899. doi: 10.1097/CCM.0000000000006216. 38502804 PMC11093432

[30] Zonneveld LEEC, van Wijk RJ, Olgers TJ, et al. Prognostic value of serial score measurements of the national early warning score, the quick sequential organ failure assessment and the systemic inflammatory response syndrome to predict clinical outcome in early sepsis. Eur J Emerg Med. 2022;29(5):348–356. doi: 10.1097/MEJ.0000000000000924. 36062434 PMC9432814

[31] Almutary A, Althunayyan S, Alenazi K, et al. National Early Warning Score (NEWS) as prognostic triage tool for septic patients. Infect Drug Resist. 2020;13:3843–3851. doi: 10.2147/IDR.S275390. 33149629 PMC7602891

[32] Faisal M, Richardson D, Scally AJ, et al. Computer-aided national early warning score to predict the risk of sepsis following emergency medical admission to hospital: a model development and external validation study. CMAJ. 2019;191(14):E382–e389. doi: 10.1503/cmaj.181418. 30962196 PMC6453675

[33] Yu H, Nie L, Liu A, et al. Combining procalcitonin with the qSOFA and sepsis mortality prediction. Medicine (Baltimore). 2019;98(23):e15981. doi: 10.1097/MD.0000000000015981. 31169735 PMC6571275

[34] Smok B, Domagalski K, Pawłowska M. Diagnostic and prognostic value of IL-6 and sTREM-1 in SIRS and sepsis in children. Mediators Inflamm. 2020;2020:8201585–8201588. doi: 10.1155/2020/8201585. 32655314 PMC7327583

[35] Harbarth S, Holeckova K, Froidevaux C, et al. Diagnostic value of procalcitonin, interleukin-6, and interleukin-8 in critically ill patients admitted with suspected sepsis. Am J Respir Crit Care Med. 2001;164(3):396–402. doi: 10.1164/ajrccm.164.3.2009052. 11500339

