# Supplementary material for: Combining host immune response biomarkers and clinical scores for early prediction of sepsis in infection patients
Source: Ann Med. 2024 Aug 30;56(1):2396569. doi: 10.1080/07853890.2024.2396569 (PMC11370677; doi:10.1080/07853890.2024.2396569)
Supplement: Supplemental Material [file IANN_A_2396569_SM5061.zip › suppl_data/Figure captions.docx]

**Figure S1.** Kaplan–Meier curves for 28-day mortality according to IL-6 (A), IL-10 (B), CRP (C), PCT (D), NEWS (E) and SIRS (F) cut-offs.

**Figure S2.** AUROC analysis for comparing IL-6, IL-10, CRP, PCT, NEWS, SIRS, MEWS with progression of infection to sepsis (A), ICU admission (B), 28-day mortality (C) and in-hospital mortality (D).

**Figure S3.** Restricted cubic splines of serum IL-6 concentrations for hazard ratios of 28-day death in all patients.

**Figure S4.** Subgroup analysis of predictive value of high IL-6 vs low IL-6 for 28-day mortality in infection patients.
