# Supplementary material for: Combining host immune response biomarkers and clinical scores for early prediction of sepsis in infection patients
Source: Ann Med. 2024 Aug 30;56(1):2396569. doi: 10.1080/07853890.2024.2396569 (PMC11370677; doi:10.1080/07853890.2024.2396569)
Supplement: Supplemental Material [file IANN_A_2396569_SM5061.zip › suppl_data/Supplementary Table_clean version.docx]

**Additional File 1**

**Contents**

**Supplementary Tables.....................................................................................................................2**

**Table S1. Logistic regression analysis for progression of infection to sepsis**

**Table S2. Bivariate IL-10 Logistic regression models for Progression of Infection to Sepsis**

**Table S3. Univariate and Multivariate Logistic Regression Analysis for ICU Admission**

**Table S4. Bivariate IL-6 Logistic Regression Models for ICU Admission**

**Table S5. Univariate and Multivariate Logistic Regression Analysis for In-hospital mortality**

**Table S6. Bivariate IL-6 Logistic Regression Models for In-hospital mortality**

**Table S7. Univariate and Multivariate Cox Regression Analysis for 28-day all-cause Mortality**

**Table S8. Outcome of patients with low/high biomarker or clinical score values**

**Table S9. AUC of patients with low/high biomarker or clinical score**

**Table S10. AUROC analysis for progression of infection to sepsis**

**Table S11. AUROC analysis for ICU admission**

**Table S12. AUROC analysis for in-hospital mortality**

**Table S13. AUROC analysis for 28-day mortality**

**Table S14. Patient subgroups stratified by IL-10 and SIRS**

**Table S15. Patient subgroups stratified by IL-6 and NEWS**

**Table S16. Patient subgroups stratified by IL-6 and SIRS**

Table S1 Logistic regression analysis for progression of infection to sepsis

| Variables | Unadjusted OR (95%CI) | *P*-value | AUC | Adjusted OR (95%CI) | *P*-value |
| --- | --- | --- | --- | --- | --- |
| IL-2 | 0.99 (0.83 - 1.16) | 0.86 |  |  | NS |
| IL-4 | 0.98 (0.83 - 1.16) | 0.85 |  |  | NS |
| IL-6 | 1.85 (1.54 - 2.22) | <0.001 | 0.701 | 1.81 (1.48 - 2.20) | <0.001 |
| IL-10 | 2.22 (1.83 - 2.69) | <0.001 | 0.733 | 2.20 (1.78 - 2.71) | <0.001 |
| INF-γ | 1.32 (1.11 - 1.56) | <0.001 | 0.585 | 1.32 (1.10 - 1.59) | 0.003 |
| TNF-α | 1.10 (0.93 - 1.30) | 0.26 |  |  | NS |
| IgA | 0.97 (0.82 - 1.14) | 0.70 |  |  | NS |
| IgG | 1.06 (0.90 - 1.25) | 0.47 |  |  | NS |
| IgE | 1.23 (1.04 - 1.45) | 0.02 | 0.572 | 1.22 (1.02 - 1.47) | 0.028 |
| IgM | 0.90 (0.76 - 1.06) | 0.19 |  |  | NS |
| C3 | 0.66 (0.55 - 0.78) | <0.001 | 0.632 | 0.72 (0.59 - 0.86) | <0.001 |
| C4 | 1.07 (0.91 - 1.26) | 0.41 |  |  | NS |
| CD4 | 0.74 (0.63 - 0.88) | <0.001 | 0.604 | 0.77 (0.64 - 0.92) | 0.004 |
| CD3 | 0.72 (0.61 - 0.85) | <0.001 | 0.606 | 0.72 (0.60 - 0.87) | <0.001 |
| CD8 | 0.82 (0.69 - 0.96) | 0.02 | 0.562 | 0.80 (0.66 - 0.96) | 0.020 |
| CD19 | 1.31 (1.11 - 1.55) | <0.001 | 0.592 | 1.34 (1.12 - 1.61) | 0.002 |
| CD4/8 | 1.00 (0.85 - 1.18) | 0.999 |  |  | NS |
| CD16/56 | 1.15 (0.98 - 1.36) | 0.09 |  |  | NS |
| CRP | 1.82 (1.52 - 2.18) | <0.001 | 0.698 | 1.72 (1.42 - 2.09) | <0.001 |
| PCT | 1.74 (1.46 - 2.08) | <0.001 | 0.680 | 1.65 (1.36 – 2.00) | <0.001 |
| NEWS | 2.00 (1.66 - 2.41) | <0.001 | 0.707 | 1.92 (1.57 - 2.34) | <0.001 |
| SIRS | 2.10 (1.71 - 2.58) | <0.001 | 0.689 | 2.15 (1.72 - 2.70) | <0.001 |
| MEWS | 1.34 (1.05 - 1.73) | 0.021 | 0.560 | 1.32 (1.00 - 1.73) | 0.050 |

Adjusted models include age, hypertension, diabetes, cardiovascular disease, liver disease and renal disease; AUC, the area under the receiver operating characteristic curve.

Table S2 Bivariate IL-10 Logistic regression models for Progression of Infection to Sepsis

| IL-10 + clinical score or biomarker | OR (95%CI) | *P*-value | AUC | NRI | *P*-value | IDI | *P*-value |
| --- | --- | --- | --- | --- | --- | --- | --- |
| IL-6 | 1.34 (1.08 - 1.66) | 0.007 | 0.753 | 0.508 (0.336 - 0.679) | <0.001 | 0.078 (0.049 - 0.108) | <0.001 |
| CRP | 1.52 (1.26 - 1.85) | <0.001 | 0.770 | 0.554 (0.383 - 0.726) | <0.001 | 0.126 (0.090 - 0.163) | <0.001 |
| PCT | 1.44 (1.19 - 1.75) | <0.001 | 0.743 | 0.667 (0.497 - 0.836) | <0.001 | 0.153 (0.114 - 0.192) | <0.001 |
| NEWS | 1.85 (1.52 - 2.26) | <0.001 | 0.789 | 0.631 (0.463 - 0.799) | <0.001 | 0.130 (0.093 - 0.167) | <0.001 |
| SIRS | 1.77 (1.43.- 2.20) | <0.001 | 0.763 | 0.553 (0.381 - 0.725) | <0.001 | 0.116 (0.081 - 0.150) | <0.001 |
| MEWS | 1.24 (0.95 - 1.63) | 0.12 | 0.740 | 0.694 (0.526 - 0.861) | <0.001 | 0.176 (0.135 - 0.218) | <0.001 |

IL, Interleukin; CRP, C-reactive protein; PCT, procalcitonin; NEWS, National Early Warning Score; SIRS, Systemic Inflammatory Response Syndrome; MEWS, Modified Early Warning Score; OR, odds ratio; CI, confidence interval; NRI, net-reclassification index; IDI, integrated discrimination improvement; AUC, the area under the receiver operating characteristic curve.

Tables S3 Univariate and Multivariate Logistic Regression Analysis for ICU Admission

| Variables | Univariate analysis  OR (95%CI) | *P*-value | AUC | Multivariate analysis  OR (95%CI) | *P*-value | AUC |
| --- | --- | --- | --- | --- | --- | --- |
| IL-6 | 2.50 (1.95 - 3.21) | <0.001 | 0.776 | 2.43 (1.86 - 3.17) | <0.001 | 0.806 |
| IL-10 | 2.42 (1.89 - 3.10) | <0.001 | 0.768 | 2.37 (1.82 - 3.08) | <0.001 | 0.773 |
| CRP | 2.77 (2.13 - 3.61) | <0.001 | 0.793 | 2.80 (2.10 - 3.74) | <0.001 | 0.835 |
| PCT | 2.06 (1.63 - 2.59) | <0.001 | 0.711 | 1.95 (1.52 - 2.51) | <0.001 | 0.745 |
| NEWS | 2.45 (1.94 - 3.08) | <0.001 | 0.767 | 2.39 (1.87 - 3.05) | <0.001 | 0.831 |
| SIRS | 2.11 (1.67 - 2.66) | <0.001 | 0.710 | 2.11 (1.64 - 2.72) | <0.001 | 0.782 |
| MEWS | 1.48 (1.10 - 1.98) | 0.009 | 0.580 | 1.53 (1.12 - 2.10) | 0.01 | 0.741 |

IL, Interleukin; CRP, C-reactive protein; PCT, procalcitonin; NEWS, National Early Warning Score; SIRS, Systemic Inflammatory Response Syndrome; MEWS, Modified Early Warning Score; OR, odds ratio; CI, confidence interval; AUC, the area under the receiver operating characteristic curve.

Table S4 Bivariate IL-6 Logistic Regression Models for ICU Admission

| IL-6 + clinical score or biomarker | OR (95%CI) | *P*-value | AUC | NRI (95%CI) | *P*-value | IDI (95%CI) | *P*-value |
| --- | --- | --- | --- | --- | --- | --- | --- |
| IL-10 | 1.87 (1.41 - 2.48) | <0.001 | 0.803 | 0.414 (0.270 - 0.557) | <0.001 | 0.046 (0.023 - 0.070) | <0.001 |
| CRP | 2.12 (1.60 - 2.86) | <0.001 | 0.820 | 0.394 (0.250 - 0.539) | <0.001 | 0.050 (0.027 - 0.074) | <0.001 |
| PCT | 1.51 (1.16 - 1.98) | 0.002 | 0.779 | 0.462 (0.319 - 0.605) | <0.001 | 0.084 (0.052 - 0.117) | <0.001 |
| NEWS | 2.41 (1.89 - 3.12) | <0.001 | 0.832 | 0.444 (0.301 - 0.587) | <0.001 | 0.074 (0.044 - 0.105) | <0.001 |
| SIRS | 1.69 (1.31 - 2.18) | <0.001 | 0.796 | 0.421 (0.280 - 0.563) | <0.001 | 0.070 (0.040 - 0.100) | <0.001 |
| MEWS | 1.48 (1.08 - 2.04) | 0.015 | 0.758 | 0.467 (0.322 - 0.611) | <0.001 | 0.100 (0.065 - 0.134) | <0.001 |

IL, Interleukin; CRP, C-reactive protein; PCT, procalcitonin; NEWS, National Early Warning Score; SIRS, Systemic Inflammatory Response Syndrome; MEWS, Modified Early Warning Score; OR, odds ratio; CI, confidence interval; NRI, net-reclassification index; IDI, integrated discrimination improvement; AUC, the area under the receiver operating characteristic curve.

Table S5 Univariate and Multivariate Logistic Regression Analysis for In-hospital mortality

| Variables | Univariate analysis  OR (95%CI) | *P*-value | AUC | Multivariate analysis  OR (95%CI) | *P*-value |
| --- | --- | --- | --- | --- | --- |
| IL-6 | 2.60 (1.82 - 3.90) | <0.001 | 0.797 | 2.36 (1.62 - 3.59) | <0.001 |
| IL-10 | 2.60 (1.82 - 3.90) | <0.001 | 0.778 | 2.45 (1.68 - 3.76) | <0.001 |
| CRP | 3.06 (2.08 - 4.78) | <0.001 | 0.782 | 3.10 (2.02 - 5.13) | <0.001 |
| PCT | 1.90 (1.39 - 2.70) | <0.001 | 0.683 | 1.66 (1.18 - 2.4) | 0.005 |
| NEWS | 2.34 (1.71 - 3.28) | <0.001 | 0.760 | 2.27 (1.63 - 3.26) | <0.001 |
| SIRS | 2.14 (1.56 - 2.97) | <0.001 | 0.724 | 2.18 (1.53 - 3.15) | <0.001 |
| MEWS | 1.43 (0.94 - 2.15) | 0.084 | 0.572 | 1.54 (0.99 - 2.39) | 0.052 |

IL, Interleukin; CRP, C-reactive protein; PCT, procalcitonin; NEWS, National Early Warning Score; SIRS, Systemic Inflammatory Response Syndrome; MEWS, Modified Early Warning Score; OR, odds ratio; CI, confidence interval; AUC, the area under the receiver operating characteristic curve.

Table S6 Bivariate IL-6 Logistic Regression Models for In-hospital mortality

| IL-6 + clinical score or biomarker | OR (95%CI) | *P*-value | AUC | NRI (95%CI) | *P*-value | IDI (95%CI) | *P*-value |
| --- | --- | --- | --- | --- | --- | --- | --- |
| IL-10 | 1.86 (1.24 - 2.91) | 0.004 | 0.807 | 0.413 (0.276 - 0.550) | <0.001 | 0.031 (0.010 - 0.052) | <0.001 |
| CRP | 2.32 (1.52 - 3.75) | <0.001 | 0.825 | 0.379 (0.245 - 0.513) | <0.001 | 0.035 (0.015 - 0.055) | <0.001 |
| PCT | 1.32 (0.92 - 1.95) | 0.150 | 0.794 | 0.416 (0.283 - 0.549) | <0.001 | 0.052 (0.028 - 0.075) | <0.001 |
| NEWS | 2.16 (1.57 - 3.07) | <0.001 | 0.828 | 0.356 (0.225 - 0.488) | <0.001 | 0.040 (0.018 - 0.061) | <0.001 |
| SIRS | 1.72 (1.21 - 2.45) | 0.003 | 0.822 | 0.409 (0.276 - 0.543) | <0.001 | 0.041 (0.019 - 0.062) | <0.001 |

IL, Interleukin; CRP, C-reactive protein; PCT, procalcitonin; NEWS, National Early Warning Score; SIRS, Systemic Inflammatory Response Syndrome; MEWS, Modified Early Warning Score; OR, odds ratio; CI, confidence interval; NRI, net-reclassification index; IDI, integrated discrimination improvement; AUC, the area under the receiver operating characteristic curve.

Table S7 Univariate and Multivariate Cox Regression Analysis for 28-day all-cause Mortality

| Variables | Univariate analysis  HR (95%CI) | *P*-value | Multivariate analysis  HR (95%CI) | *P*-value |
| --- | --- | --- | --- | --- |
| IL-6 | 2.68 (1.61 - 4.46) | <0.001 | 2.60 (1.56 - 4.33) | <0.001 |
| IL-10 | 2.06 (1.33 - 3.21) | <0.001 | 1.98 (1.27 - 3.10) | 0.003 |
| CRP | 2.63 (1.58 - 4.38) | <0.001 | 2.56 (1.51 - 4.35) | <0.001 |
| PCT | 1.68 (1.13 - 2.52) | 0.01 | 1.55 (1.02 - 2.34) | 0.039 |
| NEWS | 1.74 (1.21 - 2.49) | <0.001 | 1.80 (1.23 - 2.62) | 0.002 |
| SIRS | 1.59 (1.11 - 2.29) | 0.01 | 1.63 (1.12 - 2.39) | 0.01 |
| MEWS | 1.00 (0.61 - 1.62) | 0.999 | 1.04 (0.64 - 1.70) | 0.87 |

IL, Interleukin; CRP, C-reactive protein; PCT, procalcitonin; NEWS, National Early Warning Score; SIRS, Systemic Inflammatory Response Syndrome; MEWS, Modified Early Warning Score; HR, hazard ratio; CI, confidence interval;

Table S8 Outcome of patients with low/high biomarker or clinical score values

| Biomarker or clinical score | cut-off | population | sepsis | ICU admission | 28-day mortality | in-hospital mortality | shock | Length of hospitalisation |
| --- | --- | --- | --- | --- | --- | --- | --- | --- |
| IL-6 | <65.58 | 384 | 108(28.1%) | 46(12.0%) | 8(2.1%) | 15(3.9%) | 17(4.4%) | 7.50 [5.00;12.0] |
|  | ≥65.58 | 107 | 69(64.5%) | 51(47.7%) | 21(19.6%) | 27(25.2%) | 22(20.6%) | 11.0 [7.00;18.5] |
| IL-10 | <6.46 | 342 | 84(24.6%) | 37(10.8%) | 7(2.0%) | 12(3.5%) | 11(3.2%) | 7.00 [5.00;11.0] |
|  | ≥6.46 | 149 | 93(62.4%) | 60(40.3%) | 22(14.8%) | 30(20.1%) | 28(18.8%) | 10.0 [6.00;21.0] |
| PCT | <0.222 | 252 | 59(23.4%) | 19(7.5%) | 5(2.0%) | 8(3.1%) | 9(3.6%) | 7.00 [5.00;11.0] |
|  | ≥0.222 | 239 | 118(49.4%) | 78(32.6%) | 24(10.0%) | 34(14.2%) | 30(12.6%) | 9.00 [6.00;16.5] |
| CRP | <117.93 | 372 | 110(29.6%) | 43(11.6%) | 8(2.2%) | 15(4.0%) | 17(4.6%) | 8.00 [5.00;12.0] |
|  | ≥117.93 | 119 | 67(56.3) | 54(45.4%) | 21(17.6%) | 27(22.7%) | 22(18.5%) | 10.0 [6.00;18.5] |
| NEWS | <5 | 413 | 113(27.4%) | 50(12.1%) | 13(3.1%) | 18(4.4%) | 24(5.8%) | 8.00 [5.00;12.0] |
|  | ≥5 | 78 | 64(82.1%) | 47(60.3%) | 16(20.5%) | 24(30.8%) | 15(19.2%) | 15.0 [6.00;27.5] |
| SIRS | <2 | 341 | 88(25.8%) | 44(12.9%) | 10(2.9%) | 15(4.4%) | 13(3.8%) | 8.00 [5.00;11.0] |
|  | ≥2 | 150 | 89(59.3%) | 53(35.3%) | 19(12.7%) | 27(18.0%) | 26(17.3%) | 10.0 [6.00;19.0] |

IL, Interleukin; CRP, C-reactive protein; PCT, procalcitonin; NEWS, National Early Warning Score; SIRS, Systemic Inflammatory Response Syndrome; MEWS, Modified Early Warning Score.

Table S9 AUC of patients with low/high biomarker or clinical score

| Subgroup | AUC | | | | |
| --- | --- | --- | --- | --- | --- |
|  | IL-6 | IL-10 | NEWS | SIRS | CRP |
| IL-6 | | | | | |
| <65.58 |  | 0.753 | 0.729 | 0.742 | 0.765 |
| ≥65.58 |  | 0.682 | 0.742 | 0.568 | 0.818 |
| IL-10 | | | | | |
| <6.46 | 0.747 |  | 0.752 | 0.632 | 0.716 |
| ≥6.46 | 0.780 |  | 0.693 | 0.655 | 0.745 |
| NEWS | | | | | |
| <5 | 0.819 | 0.788 |  | 0.718 | 0.722 |
| ≥5 | 0.829 | 0.702 |  | 0.592 | 0.837 |
| SIRS | | | | | |
| <2 | 0.859 | 0.762 | 0.729 |  | 0.771 |
| ≥2 | 0.749 | 0.735 | 0.671 |  | 0.712 |
| CRP | | | | | |
| <117.93 | 0.846 | 0.692 | 0.657 | 0.755 |  |
| ≥117.93 | 0.704 | 0.745 | 0.749 | 0.571 |  |

IL, Interleukin; CRP, C-reactive protein; PCT, procalcitonin; NEWS, National Early Warning Score; SIRS, Systemic Inflammatory Response Syndrome; AUC, the area under the receiver operating characteristic curve.

Table S10 AUROC analysis for progression of infection to sepsis

|  | AUC | SEN | SPE | PLR | NLR | PPV | NPV | cut-off | OR |
| --- | --- | --- | --- | --- | --- | --- | --- | --- | --- |
| IL-6 | 0.701(0.652-0.751) | 0.55(0.48-0.62) | 0.76(0.72-0.81) | 2.33(1.83-2.96) | 0.59(0.50-0.70) | 0.57(0.49-0.64) | 0.75(0.70-0.80) | 33.245 | 3.9 |
| IL-10 | 0.733(0.685-0.780) | 0.73(0.66-0.79) | 0.63(0.58-0.69) | 1.99(1.68-2.36) | 0.43(0.33-0.55) | 0.53(0.47-0.59) | 0.81(0.76-0.86) | 5.025 | 4.7 |
| CRP | 0.698(0.651-0.745) | 0.64(0.57-0.72) | 0.65(0.60-0.70) | 1.84(1.53-2.21) | 0.55(0.44-0.68) | 0.51(0.44-0.57) | 0.76(0.71-0.82) | 52.315 | 3.4 |
| PCT | 0.680(0.632-0.728) | 0.83(0.78-0.89) | 0.49(0.44-0.55) | 1.63(1.44-1.85) | 0.35(0.25-0.49) | 0.48(0.42-0.54) | 0.84(0.78-0.89) | 0.09 | 4.7 |
| NEWS | 0.707(0.658-0.757) | 0.36(0.29-0.43) | 0.96(0.93-0.98) | 0.82(0.74-0.91) | 0.73(0.68-0.77) | 8.11(4.69-14.03) | 0.67(0.60-0.75) | 5 | 12.1 |
| SIRS | 0.689(0.641-0.736) | 0.50(0.43-0.58) | 0.81(0.76-0.85) | 0.59(0.51-0.67) | 0.74(0.70-0.79) | 2.59(1.98-3.39) | 0.62(0.53-0.73) | 2 | 4.2 |
| MEWS | 0.560(0.510-0.609) | 0.33(0.26-0.40) | 0.79(0.75-0.84) | 0.47(0.38-0.56) | 0.68(0.63-0.72) | 1.56(1.15-2.11) | 0.85(0.76-0.96) | 3 | 1.8 |

IL, Interleukin; CRP, C-reactive protein; PCT, procalcitonin; NEWS, National Early Warning Score; SIRS, Systemic Inflammatory Response Syndrome; MEWS, Modified Early Warning Score; AUC, the area under the receiver operating characteristic curve. SEN, sensitivity; SPE, specificity; PLR, positive likelihood ratio; NLR, negative likelihood ratio; PPV, positive predictive value; NPV, negative predictive value; OR, odds ratio.

Table S11 AUROC analysis for ICU admission

|  | AUC | SEN | SPE | PLR | NLR | PPV | NPV | cut-off | OR |
| --- | --- | --- | --- | --- | --- | --- | --- | --- | --- |
| IL-6 | 0.776(0.723-0.829) | 0.85(0.77-0.92) | 0.57(0.52-0.62) | 1.97(1.71-2.27) | 0.27(0.17-0.44) | 0.33(0.27-0.39) | 0.94(0.91-0.97) | 16.81 | 7.3 |
| IL-10 | 0.768(0.716-0.820) | 0.74(0.66-0.83) | 0.67(0.62-0.71) | 2.22(1.85-2.66) | 0.39(0.28-0.55) | 0.35(0.29-0.42) | 0.91(0.88-0.95) | 5.555 | 5.7 |
| CRP | 0.793(0.746-0.841) | 0.80(0.73-0.88) | 0.69(0.64-0.73) | 2.58(2.16-3.07) | 0.29(0.19-0.43) | 0.39(0.32-0.46) | 0.93(0.91-0.96) | 65.325 | 9 |
| PCT | 0.711(0.655-0.766) | 0.81(0.74-0.89) | 0.59(0.54-0.64) | 1.98(1.70-2.30) | 0.32(0.21-0.48) | 0.33(0.27-0.39) | 0.93(0.90-0.96) | 0.216 | 6.3 |
| NEWS | 0.767(0.712-0.823) | 0.49(0.39-0.58) | 0.92(0.90-0.95) | 6.16(4.15-9.15) | 0.56(0.46-0.68) | 0.60(0.49-0.71) | 0.88(0.85-0.91) | 5 | 11 |
| SIRS | 0.710(0.658-0.762) | 0.90(0.84-0.96) | 0.42(0.37-0.47) | 1.54(1.39-1.72) | 0.25(0.14-0.45) | 0.28(0.23-0.33) | 0.94(0.91-0.98) | 1 | 6.3 |
| MEWS | 0.580(0.519-0.641) | 0.37(0.28-0.47) | 0.78(0.74-0.82) | 1.66(1.21-2.28) | 0.81(0.69-0.95) | 0.29(0.21-0.37) | 0.83(0.80-0.87) | 3 | 2.1 |

IL, Interleukin; CRP, C-reactive protein; PCT, procalcitonin; NEWS, National Early Warning Score; SIRS, Systemic Inflammatory Response Syndrome; MEWS, Modified Early Warning Score; AUC, the area under the receiver operating characteristic curve. SEN, sensitivity; SPE, specificity; PLR, positive likelihood ratio; NLR, negative likelihood ratio; PPV, positive predictive value; NPV, negative predictive value; OR, odds ratio.

Table S12 AUROC analysis for in-hospital mortality

|  | AUC | SEN | SPE | PLR | NLR | PPV | NPV | cut-off | OR |
| --- | --- | --- | --- | --- | --- | --- | --- | --- | --- |
| IL-6 | 0.797(0.725-0.869) | 0.79(0.66-0.91) | 0.70(0.66-0.74) | 2.61(2.11-3.23) | 0.31(0.17-0.55) | 0.20(0.14-0.26) | 0.97(0.95-0.99) | 34.195 | 8.5 |
| IL-10 | 0.778(0.701-0.854) | 0.74(0.61-0.87) | 0.71(0.67-0.76) | 2.57(2.04-3.24) | 0.37(0.22-0.61) | 0.19(0.13-0.26) | 0.97(0.95-0.99) | 6.265 | 7 |
| CRP | 0.782(0.721-0.843) | 0.86(0.75-0.96) | 0.63(0.59-0.68) | 2.33(1.96-2.77) | 0.23(0.11-0.48) | 0.18(0.13-0.23) | 0.98(0.96-1.00) | 65.325 | 10.3 |
| PCT | 0.683(0.604-0.763) | 0.71(0.58-0.85) | 0.64(0.60-0.69) | 2.00(1.60-2.52) | 0.44(0.27-0.72) | 0.16(0.11-0.21) | 0.96(0.94-0.98) | 0.5 | 4.5 |
| NEWS | 0.760(0.679-0.840) | 0.57(0.42-0.72) | 0.88(0.85-0.91) | 4.75(3.31-6.83) | 0.49(0.34-0.69) | 0.31(0.21-0.41) | 0.96(0.94-0.98) | 5 | 9.8 |
| SIRS | 0.724(0.656-0.793) | 0.64(0.50-0.79) | 0.73(0.69-0.77) | 2.35(1.79-3.08) | 0.49(0.33-0.74) | 0.18(0.12-0.24) | 0.96(0.93-0.98) | 2 | 4.8 |

IL, Interleukin; CRP, C-reactive protein; PCT, procalcitonin; NEWS, National Early Warning Score; SIRS, Systemic Inflammatory Response Syndrome; MEWS, Modified Early Warning Score; AUC, the area under the receiver operating characteristic curve. SEN, sensitivity; SPE, specificity; PLR, positive likelihood ratio; NLR, negative likelihood ratio; PPV, positive predictive value; NPV, negative predictive value; OR, odds ratio.

Table S13 AUROC analysis for 28-day mortality

|  | AUC | SEN | SPE | PLR | NLR | PPV | NPV | cut-off | OR |
| --- | --- | --- | --- | --- | --- | --- | --- | --- | --- |
| IL-6 | 0.839(0.774-0.903) | 0.72(0.56-0.89) | 0.81(0.78-0.85) | 3.89(2.90-5.22) | 0.34(0.19-0.61) | 0.20(0.12-0.27) | 0.98(0.97-0.99) | 65.575 | 11.5 |
| IL-10 | 0.787(0.695-0.879) | 0.76(0.60-0.91) | 0.73(0.68-0.77) | 2.76(2.14-3.56) | 0.33(0.17-0.64) | 0.15(0.09-0.21) | 0.98(0.97-1.00) | 6.46 | 8.3 |
| CRP | 0.791(0.715-0.867) | 0.72(0.56-0.89) | 0.79(0.75-0.83) | 3.41(2.57-4.54) | 0.35(0.19-0.63) | 0.18(0.11-0.25) | 0.98(0.96-0.99) | 117.93 | 9.8 |
| PCT | 0.685(0.582-0.788) | 0.83(0.69-0.97) | 0.54(0.49-0.58) | 1.78(1.47-2.16) | 0.32(0.15-0.72) | 0.10(0.06-0.14) | 0.98(0.96-1.00) | 0.222 | 5.5 |
| NEWS | 0.750(0.652-0.847) | 0.55(0.37-0.73) | 0.87(0.84-0.90) | 4.11(2.75-6.14) | 0.52(0.35-0.78) | 0.21(0.12-0.30) | 0.97(0.95-0.99) | 5 | 7.9 |
| SIRS | 0.720(0.639-0.799) | 0.66(0.48-0.83) | 0.72(0.68-0.76) | 2.31(1.71-3.12) | 0.48(0.29-0.80) | 0.13(0.07-0.18) | 0.97(0.95-0.99) | 2 | 4.8 |

IL, Interleukin; CRP, C-reactive protein; PCT, procalcitonin; NEWS, National Early Warning Score; SIRS, Systemic Inflammatory Response Syndrome; MEWS, Modified Early Warning Score; AUC, the area under the receiver operating characteristic curve. SEN, sensitivity; SPE, specificity; PLR, positive likelihood ratio; NLR, negative likelihood ratio; PPV, positive predictive value; NPV, negative predictive value; OR, odds ratio.

Table S14 Patient subgroups stratified by IL-10 and SIRS

| Patient subgroups | IL-10 | SIRS | IL-10 | SIRS | IL-10 | SIRS | IL-10 | SIRS |
| --- | --- | --- | --- | --- | --- | --- | --- | --- |
|  | <5.03 | <2 | <5.03 | ≥2 | ≥5.03 | <2 | ≥5.03 | ≥2 |
| Population *N* (%) | 203 (41.3%) | | 44 (9.0%) | | 138 (28.1%) | | 106 (21.6%) | |
| Sepsis *N* (%) | 33 (16.3%) | | 15 (34.1%) | | 55 (39.9%) | | 74 (69.8%) | |
| Shock N (%) | 6 (3.0%) | | 1 (2.3%) | | 7 (5.1%) | | 25 (23.6%) | |
| ICU admission *N* (%) | 12 (5.9%) | | 7 (15.9%) | | 32 (23.2%) | | 46 (43.4%) | |
| 28-day mortality *N* (%) | 2 (1.0%) | | 2 (4.5%) | | 8 (5.8%) | | 17 (16.0%) | |
| Hospital mortality *N* (%) | 2 (1.0%) | | 4 (9.1%) | | 13 (9.4%) | | 23 (21.7%) | |
| Hospital LOS, day | 7 [5 - 10] | | 9 [6 - 13] | | 9 [6 - 12] | | 12 [6 - 22] | |

IL, Interleukin; SIRS, Systemic Inflammatory Response Syndrome.

Table S15 Patient subgroups stratified by IL-6 and NEWS

| Patient subgroups | IL-6 | NEWS | IL-6 | NEWS | IL-6 | NEWS | IL-6 | NEWS |
| --- | --- | --- | --- | --- | --- | --- | --- | --- |
|  | <33.25 | <5 | <33.25 | ≥5 | ≥33.25 | <5 | ≥33.25 | ≥5 |
| Population *N* (%) | 283 (57.6%) | | 37 (7.5%) | | 130 (26.5%) | | 41 (8.3%) | |
| Sepsis *N* (%) | 55 (19.4%) | | 25 (67.6%) | | 58 (44.6%) | | 39 (95.1%) | |
| Shock N (%) | 4 (1.4%) | | 5 (13.5%) | | 20 (15.4%) | | 10 (24.4%) | |
| ICU admission *N* (%) | 15 (5.3%) | | 17 (45.9%) | | 35 (26.9%) | | 30 (73.1%) | |
| 28-day mortality *N* (%) | 2 (0.7%) | | 3 (8.1%) | | 11 (8.5%) | | 13 (31.7%) | |
| Hospital mortality *N* (%) | 2 (0.7%) | | 7 (18.9%) | | 16 (12.3%) | | 17 (41.5%) | |
| Hospital LOS, day | 7 [5 - 10] | | 13 [5 - 31] | | 10 [7 - 15] | | 15 [7 - 24] | |

IL, Interleukin; NEWS, National Early Warning Score.

Table S16 Patient subgroups stratified by IL-6 and SIRS

| Patient subgroups | IL-6 | SIRS | IL-6 | SIRS | IL-6 | SIRS | IL-6 | SIRS |
| --- | --- | --- | --- | --- | --- | --- | --- | --- |
|  | <33.25 | <2 | <33.25 | ≥2 | ≥33.25 | <2 | ≥33.25 | ≥2 |
| Population *N* (%) | 256 (52.1%) | | 64 (13.0%) | | 85 (17.3%) | | 86 (17.5%) | |
| Sepsis *N* (%) | 52 (20.3%) | | 28 (43.8%) | | 36 (42.4%) | | 61 (70.9%) | |
| Shock N (%) | 4 (1.6%) | | 5 (7.8%) | | 9 (10.6%) | | 21 (24.4%) | |
| ICU admission *N* (%) | 18 (7.9%) | | 14 (21.9%) | | 26 (30.6%) | | 39 (45.3%) | |
| 28-day mortality *N* (%) | 3 (1.2%) | | 2 (3.1%) | | 7 (8.2%) | | 17 (19.8%) | |
| Hospital mortality *N* (%) | 5 (2.0%) | | 4 (6.3%) | | 10 (11.8%) | | 23 (26.7%) | |
| Hospital LOS, day | 7 [5 - 10] | | 9 [5 - 17] | | 10 [7 - 16] | | 11 [7 - 21] | |

IL, Interleukin; SIRS, Systemic Inflammatory Response Syndrome.
