# Supplementary figures and images for: Combining host immune response biomarkers and clinical scores for early prediction of sepsis in infection patients
Source: Ann Med. 2024 Aug 30;56(1):2396569. doi: 10.1080/07853890.2024.2396569 (PMC11370677; doi:10.1080/07853890.2024.2396569)

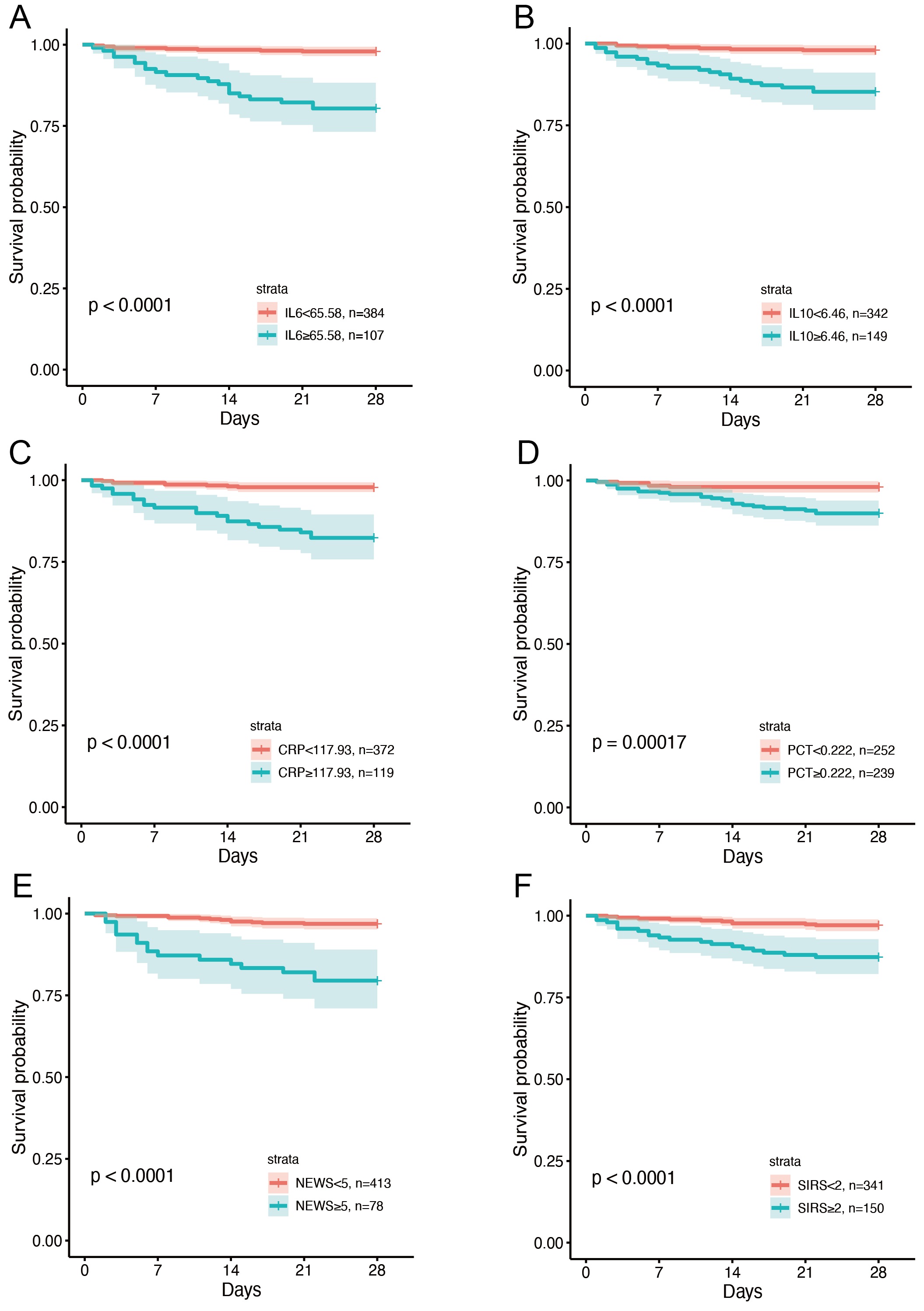

Supplement: Supplemental Material [file IANN_A_2396569_SM5061.zip › suppl_data/Fig 1S KM_single.jpg]

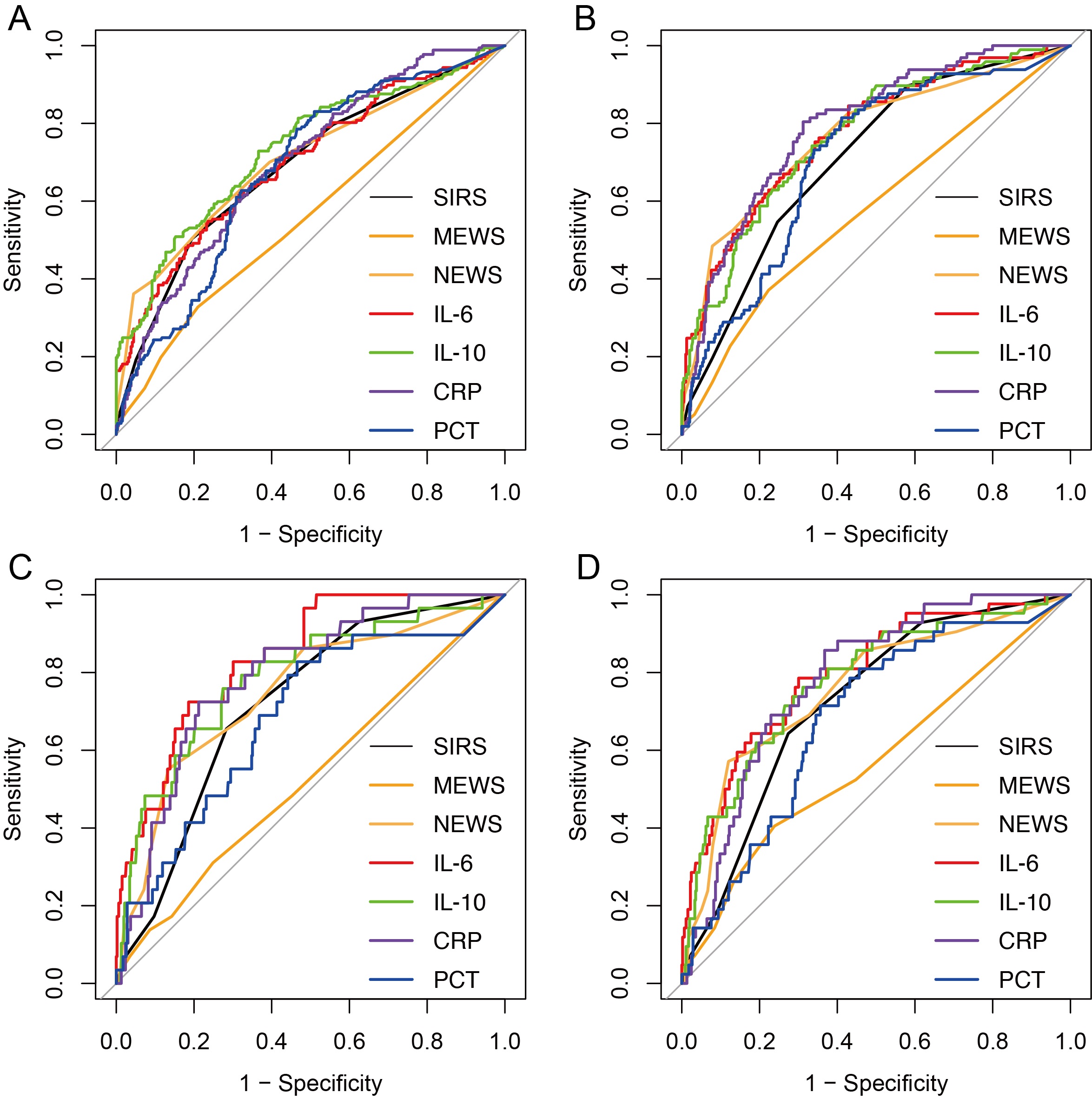

Supplement: Supplemental Material [file IANN_A_2396569_SM5061.zip › suppl_data/Fig 2S AUC.jpg]

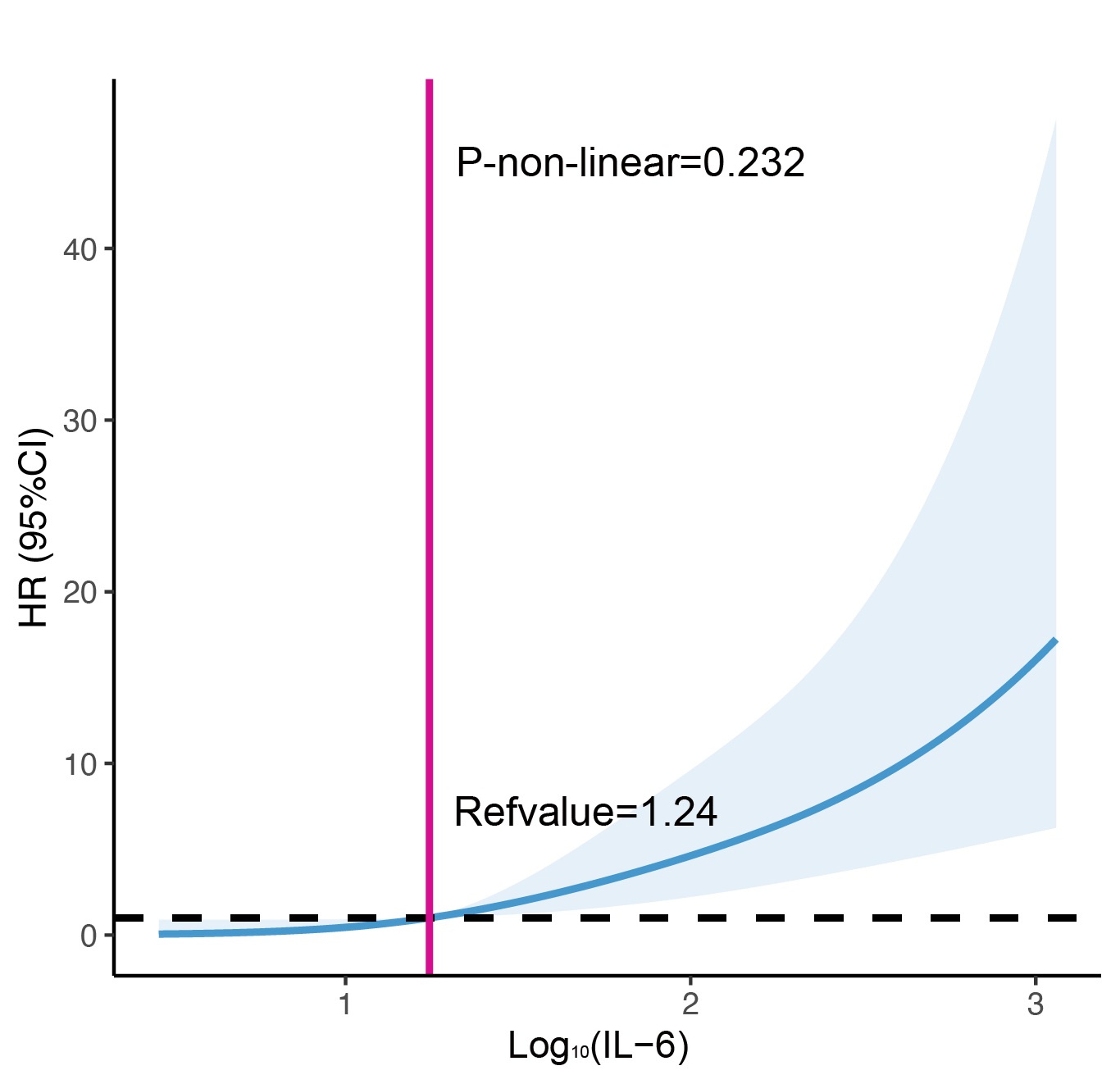

Supplement: Supplemental Material [file IANN_A_2396569_SM5061.zip › suppl_data/Fig 3S RCS.jpg]

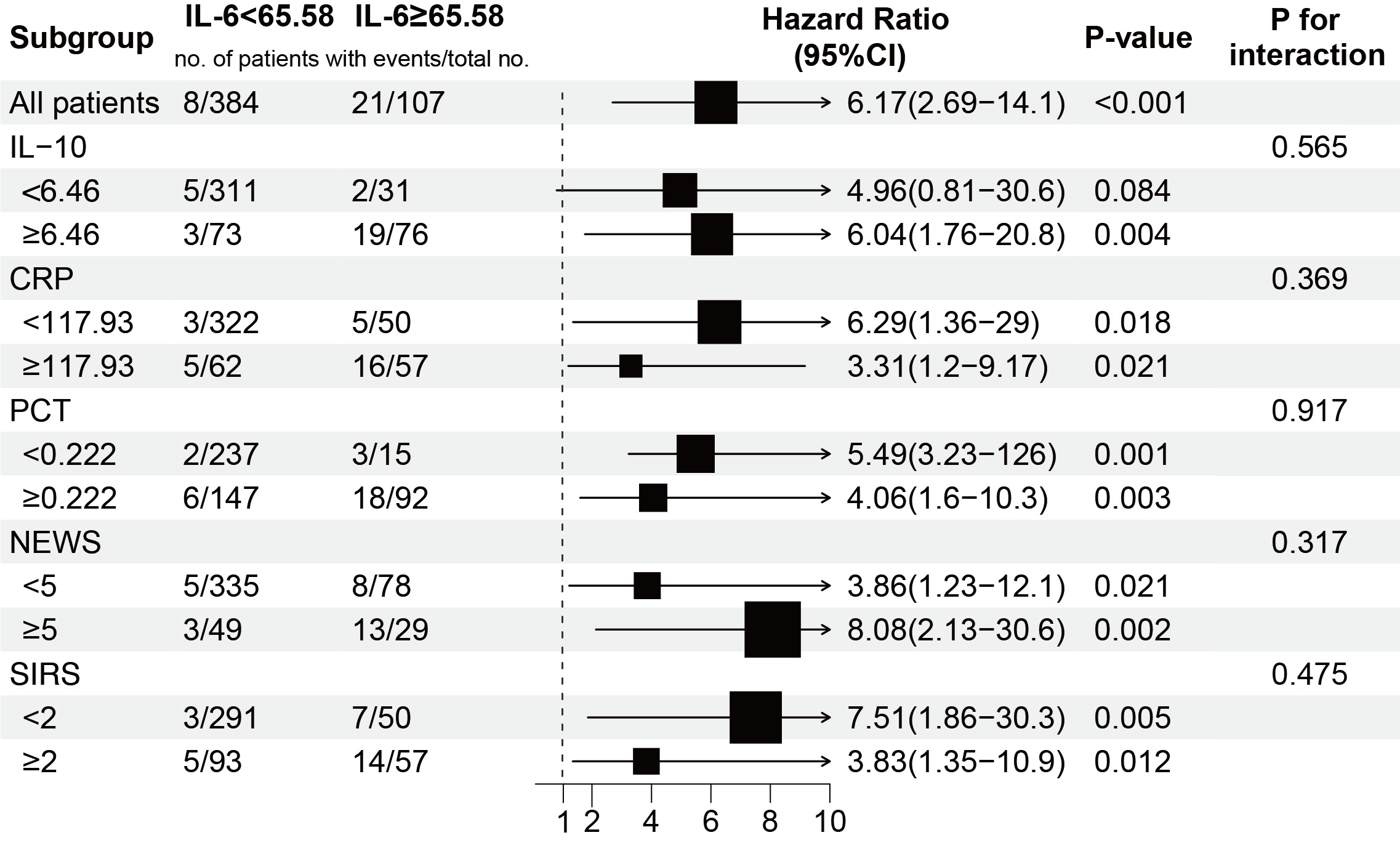

Supplement: Supplemental Material [file IANN_A_2396569_SM5061.zip › suppl_data/Fig 4S Forest Subgroup.jpg]
